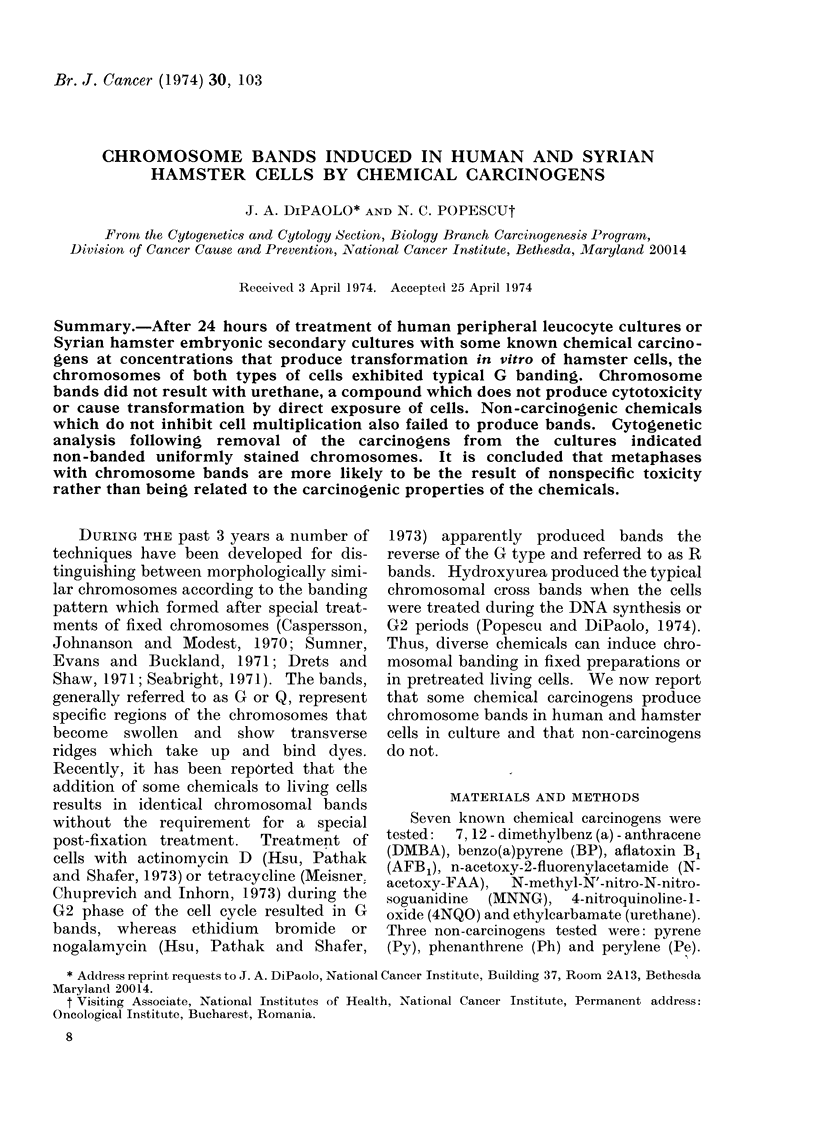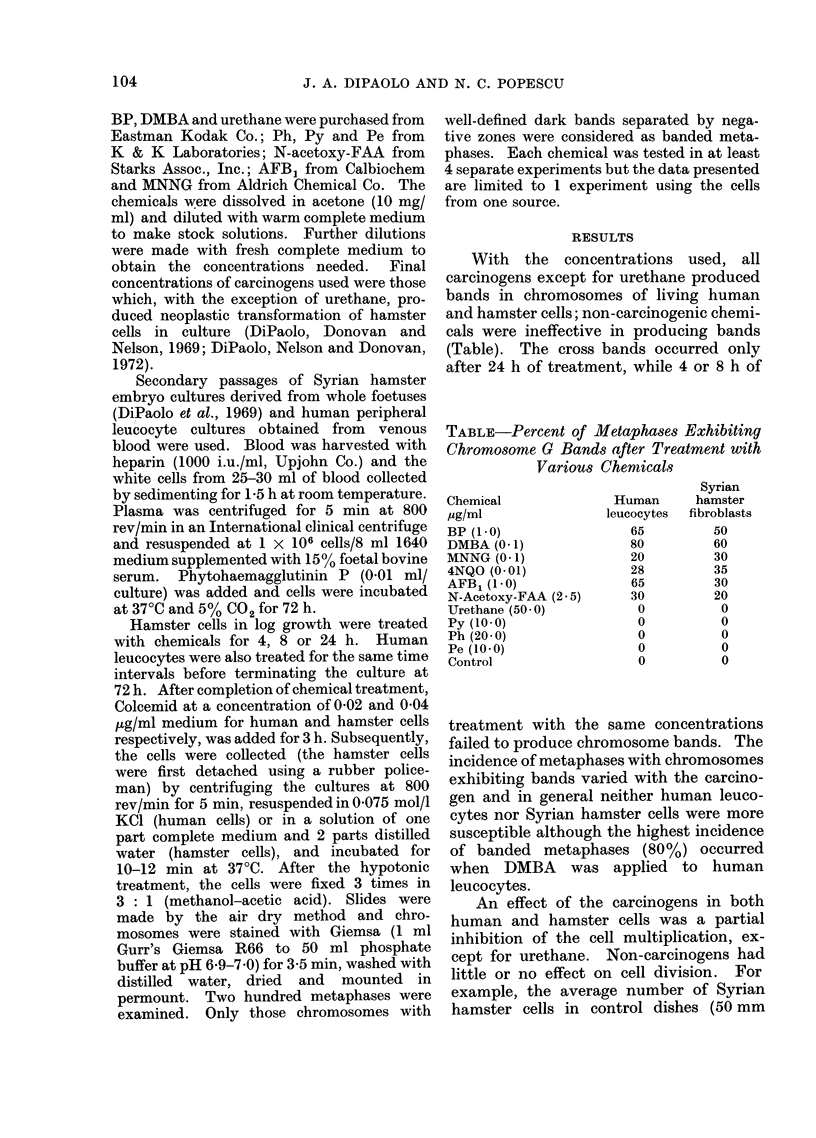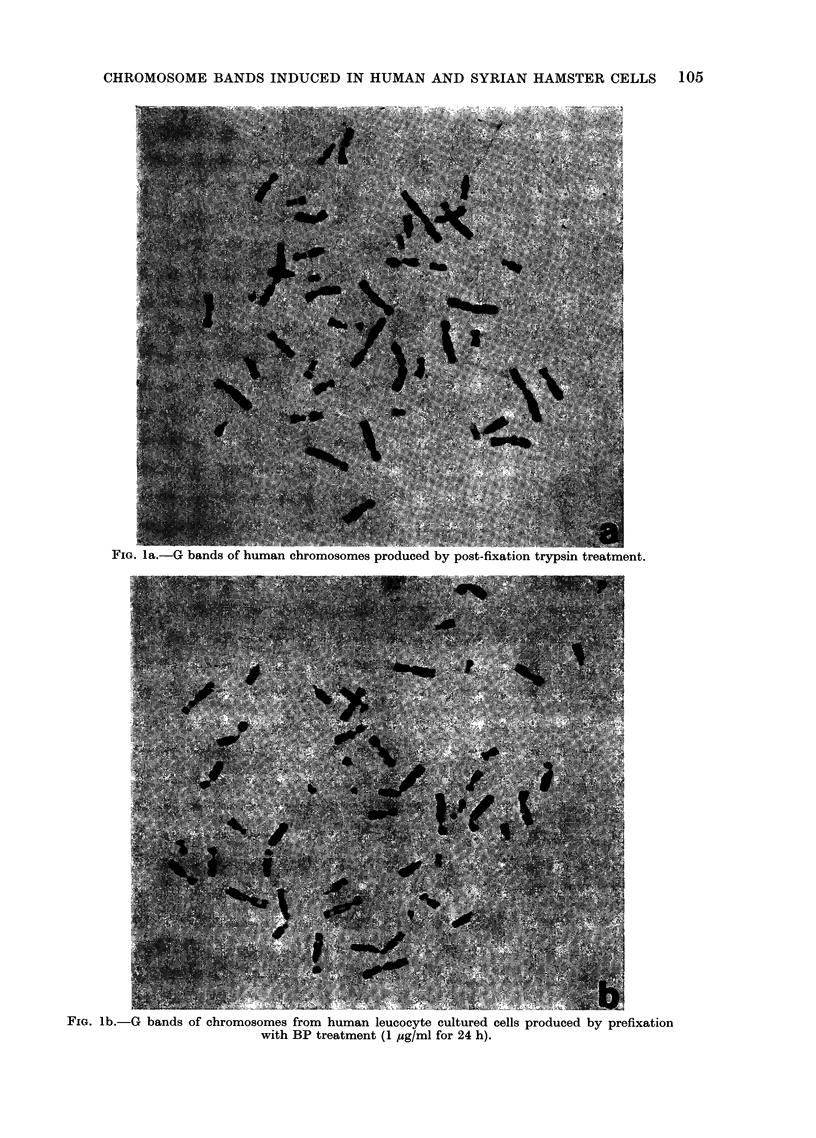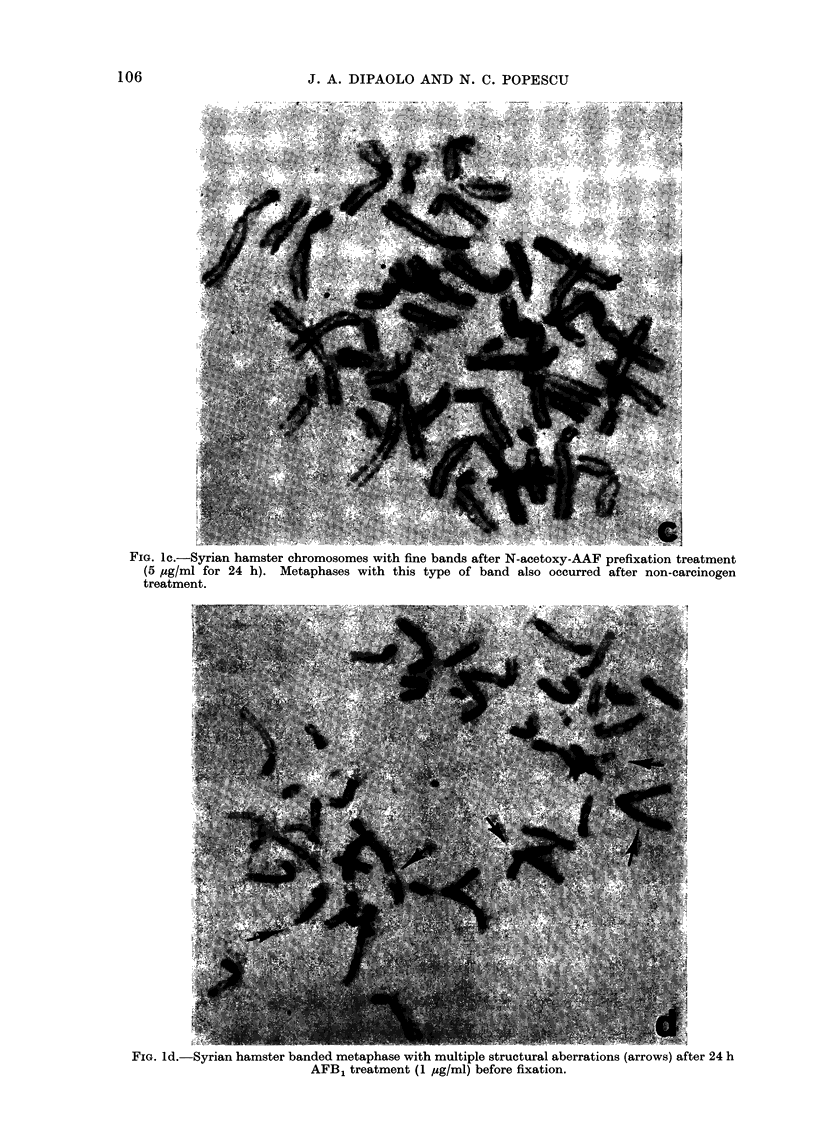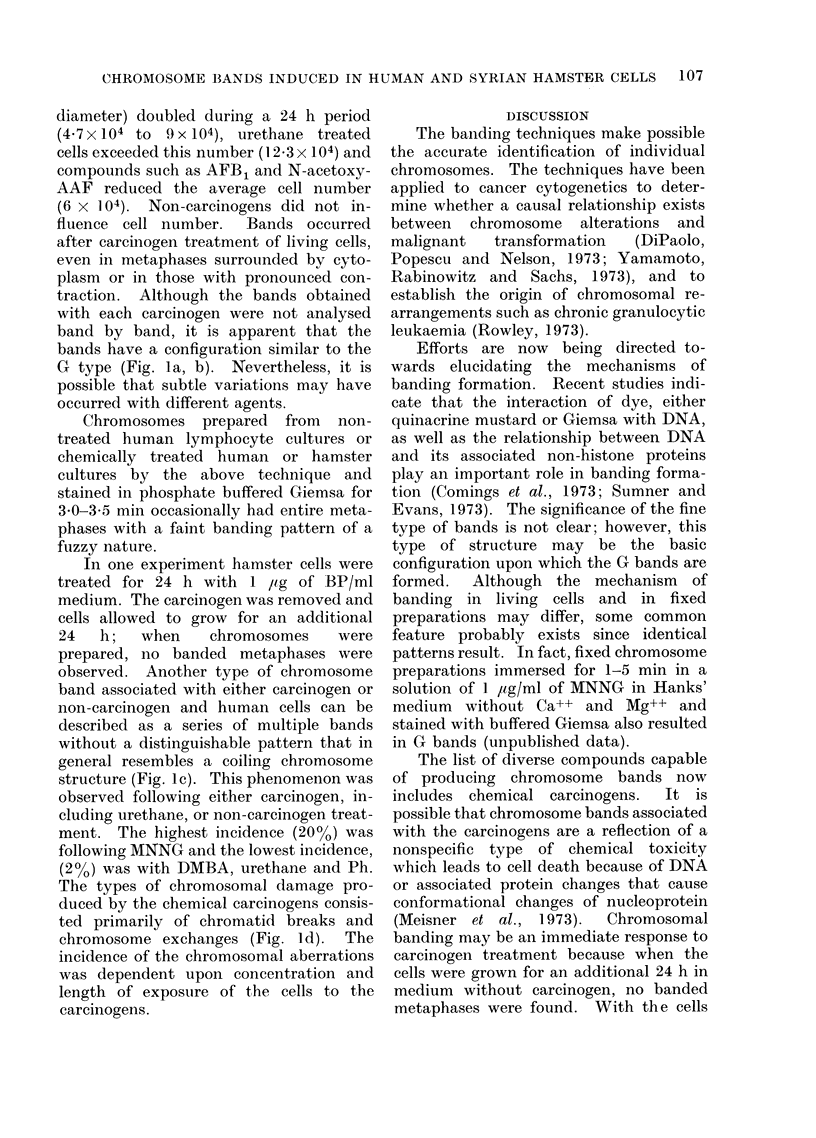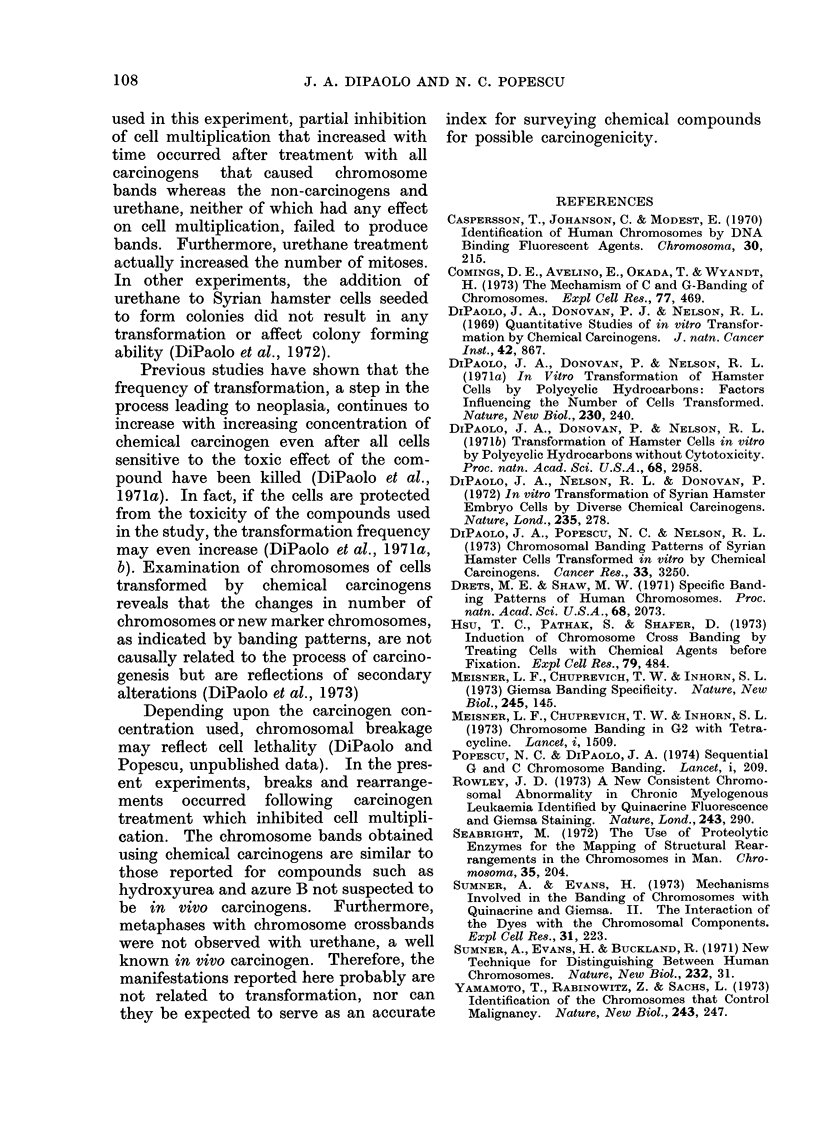# Chromosome Bands Induced in Human and Syrian Hamster Cells by Chemical Carcinogens

**DOI:** 10.1038/bjc.1974.120

**Published:** 1974-08

**Authors:** J. A. DiPaolo, N. C. Popescu

## Abstract

**Images:**


*Br. J. Cancer* (1974) **30**, 103

### CHROMOSOME BANDS INDUCED IN HUMAN AND SYRIAN HAMSTER CELLS BY CHEMICAL CARCINOGENS

J. A. DiPAOLO* AND N. C. POPESCU†

*From the Cytogenetics and Cytology Section, Biology Branch Carcinogenesis Program, Division of Cancer Cause and Prevention, National Cancer Institute, Bethesda, Maryland* 20014

Received 3 April 1974. Accepted 25 April 1974

**Summary.—After 24 hours of treatment of human peripheral leucocyte cultures or Syrian hamster embryonic secondary cultures with some known chemical carcinogens at concentrations that produce transformation *in vitro* of hamster cells, the chromosomes of both types of cells exhibited typical G banding. Chromosome bands did not result with urethane, a compound which does not produce cytotoxicity or cause transformation by direct exposure of cells. Non-carcinogenic chemicals which do not inhibit cell multiplication also failed to produce bands. Cytogenetic analysis following removal of the carcinogens from the cultures indicated non-banded uniformly stained chromosomes. It is concluded that metaphases with chromosome bands are more likely to be the result of nonspecific toxicity rather than being related to the carcinogenic properties of the chemicals.**

DURING THE past 3 years a number of techniques have been developed for distinguishing between morphologically similar chromosomes according to the banding pattern which formed after special treatments of fixed chromosomes (Caspersson, Johnanson and Modest, 1970; Sumner, Evans and Buckland, 1971; Drets and Shaw, 1971; Seabright, 1971). The bands, generally referred to as G or Q, represent specific regions of the chromosomes that become swollen and show transverse ridges which take up and bind dyes. Recently, it has been reported that the addition of some chemicals to living cells results in identical chromosomal bands without the requirement for a special post-fixation treatment. Treatment of cells with actinomycin D (Hsu, Pathak and Shafer, 1973) or tetracycline (Meisner, Chuprevich and Inhorn, 1973) during the G2 phase of the cell cycle resulted in G bands, whereas ethidium bromide or nogalamycin (Hsu, Pathak and Shafer, 1973) apparently produced bands the reverse of the G type and referred to as R bands. Hydroxyurea produced the typical chromosomal cross bands when the cells were treated during the DNA synthesis or G2 periods (Popescu and DiPaolo, 1974). Thus, diverse chemicals can induce chromosomal banding in fixed preparations or in pretreated living cells. We now report that some chemical carcinogens produce chromosome bands in human and hamster cells in culture and that non-carcinogens do not.

### MATERIALS AND METHODS

Seven known chemical carcinogens were tested: 7,12-dimethylbenz(a)-anthracene (DMBA), benzo(a)pyrene (BP), aflatoxin $B_1$ ($AFB_1$), n-acetoxy-2-fluorenylacetamide (N-acetoxy-FAA), N-methyl-N′-nitro-N-nitrosoguanidine (MNNG), 4-nitroquinoline-1-oxide (4NQO) and ethylcarbamate (urethane). Three non-carcinogens tested were: pyrene (Py), phenanthrene (Ph) and perylene (Pe).

* Address reprint requests to J. A. DiPaolo, National Cancer Institute, Building 37, Room 2A13, Bethesda Maryland 20014.

† Visiting Associate, National Institutes of Health, National Cancer Institute, Permanent address: Oncological Institute, Bucharest, Romania.

BP, DMBA and urethane were purchased from Eastman Kodak Co.; Ph, Py and Pe from K & K Laboratories; N-acetoxy-FAA from Starks Assoc., Inc.; $AFB_1$ from Calbiochem and MNNG from Aldrich Chemical Co. The chemicals were dissolved in acetone (10 mg/ml) and diluted with warm complete medium to make stock solutions. Further dilutions were made with fresh complete medium to obtain the concentrations needed. Final concentrations of carcinogens used were those which, with the exception of urethane, produced neoplastic transformation of hamster cells in culture (DiPaolo, Donovan and Nelson, 1969; DiPaolo, Nelson and Donovan, 1972).

Secondary passages of Syrian hamster embryo cultures derived from whole foetuses (DiPaolo *et al.*, 1969) and human peripheral leucocyte cultures obtained from venous blood were used. Blood was harvested with heparin (1000 i.u./ml, Upjohn Co.) and the white cells from 25–30 ml of blood collected by sedimenting for 1·5 h at room temperature. Plasma was centrifuged for 5 min at 800 rev/min in an International clinical centrifuge and resuspended at $1 \times 10^6$ cells/8 ml 1640 medium supplemented with 15% foetal bovine serum. Phytohaemagglutinin P (0·01 ml/culture) was added and cells were incubated at 37°C and 5% $CO_2$ for 72 h.

Hamster cells in log growth were treated with chemicals for 4, 8 or 24 h. Human leucocytes were also treated for the same time intervals before terminating the culture at 72 h. After completion of chemical treatment, Colcemid at a concentration of 0·02 and 0·04 μg/ml medium for human and hamster cells respectively, was added for 3 h. Subsequently, the cells were collected (the hamster cells were first detached using a rubber policeman) by centrifuging the cultures at 800 rev/min for 5 min, resuspended in 0·075 mol/l KCl (human cells) or in a solution of one part complete medium and 2 parts distilled water (hamster cells), and incubated for 10–12 min at 37°C. After the hypotonic treatment, the cells were fixed 3 times in 3 : 1 (methanol–acetic acid). Slides were made by the air dry method and chromosomes were stained with Giemsa (1 ml Gurr's Giemsa R66 to 50 ml phosphate buffer at pH 6·9–7·0) for 3·5 min, washed with distilled water, dried and mounted in permount. Two hundred metaphases were examined. Only those chromosomes with well-defined dark bands separated by negative zones were considered as banded metaphases. Each chemical was tested in at least 4 separate experiments but the data presented are limited to 1 experiment using the cells from one source.

### RESULTS

With the concentrations used, all carcinogens except for urethane produced bands in chromosomes of living human and hamster cells; non-carcinogenic chemicals were ineffective in producing bands (Table). The cross bands occurred only after 24 h of treatment, while 4 or 8 h of treatment with the same concentrations failed to produce chromosome bands. The incidence of metaphases with chromosomes exhibiting bands varied with the carcinogen and in general neither human leucocytes nor Syrian hamster cells were more susceptible although the highest incidence of banded metaphases (80%) occurred when DMBA was applied to human leucocytes.

TABLE—*Percent of Metaphases Exhibiting Chromosome G Bands after Treatment with Various Chemicals*

| Chemical μg/ml | Human leucocytes | Syrian hamster fibroblasts |
|---|---|---|
| BP (1·0) | 65 | 50 |
| DMBA (0·1) | 80 | 60 |
| MNNG (0·1) | 20 | 30 |
| 4NQO (0·01) | 28 | 35 |
| $AFB_1$ (1·0) | 65 | 30 |
| N-Acetoxy-FAA (2·5) | 30 | 20 |
| Urethane (50·0) | 0 | 0 |
| Py (10·0) | 0 | 0 |
| Ph (20·0) | 0 | 0 |
| Pe (10·0) | 0 | 0 |
| Control | 0 | 0 |

An effect of the carcinogens in both human and hamster cells was a partial inhibition of the cell multiplication, except for urethane. Non-carcinogens had little or no effect on cell division. For example, the average number of Syrian hamster cells in control dishes (50 mm

FIG. 1a.—G bands of human chromosomes produced by post-fixation trypsin treatment.

FIG. 1b.—G bands of chromosomes from human leucocyte cultured cells produced by prefixation with BP treatment (1 $\mu$g/ml for 24 h).

FIG. 1c.—Syrian hamster chromosomes with fine bands after N-acetoxy-AAF prefixation treatment (5 $\mu$g/ml for 24 h). Metaphases with this type of band also occurred after non-carcinogen treatment.

FIG. 1d.—Syrian hamster banded metaphase with multiple structural aberrations (arrows) after 24 h $AFB_1$ treatment (1 $\mu$g/ml) before fixation.

diameter) doubled during a 24 h period ($4{\cdot}7 \times 10^4$ to $9 \times 10^4$), urethane treated cells exceeded this number ($12{\cdot}3 \times 10^4$) and compounds such as $AFB_1$ and N-acetoxy-AAF reduced the average cell number ($6 \times 10^4$). Non-carcinogens did not influence cell number. Bands occurred after carcinogen treatment of living cells, even in metaphases surrounded by cytoplasm or in those with pronounced contraction. Although the bands obtained with each carcinogen were not analysed band by band, it is apparent that the bands have a configuration similar to the G type (Fig. 1a, b). Nevertheless, it is possible that subtle variations may have occurred with different agents.

Chromosomes prepared from non-treated human lymphocyte cultures or chemically treated human or hamster cultures by the above technique and stained in phosphate buffered Giemsa for 3·0–3·5 min occasionally had entire metaphases with a faint banding pattern of a fuzzy nature.

In one experiment hamster cells were treated for 24 h with 1 $\mu$g of BP/ml medium. The carcinogen was removed and cells allowed to grow for an additional 24 h; when chromosomes were prepared, no banded metaphases were observed. Another type of chromosome band associated with either carcinogen or non-carcinogen and human cells can be described as a series of multiple bands without a distinguishable pattern that in general resembles a coiling chromosome structure (Fig. 1c). This phenomenon was observed following either carcinogen, including urethane, or non-carcinogen treatment. The highest incidence (20%) was following MNNG and the lowest incidence, (2%) was with DMBA, urethane and Ph. The types of chromosomal damage produced by the chemical carcinogens consisted primarily of chromatid breaks and chromosome exchanges (Fig. 1d). The incidence of the chromosomal aberrations was dependent upon concentration and length of exposure of the cells to the carcinogens.

### DISCUSSION

The banding techniques make possible the accurate identification of individual chromosomes. The techniques have been applied to cancer cytogenetics to determine whether a causal relationship exists between chromosome alterations and malignant transformation (DiPaolo, Popescu and Nelson, 1973; Yamamoto, Rabinowitz and Sachs, 1973), and to establish the origin of chromosomal rearrangements such as chronic granulocytic leukaemia (Rowley, 1973).

Efforts are now being directed towards elucidating the mechanisms of banding formation. Recent studies indicate that the interaction of dye, either quinacrine mustard or Giemsa with DNA, as well as the relationship between DNA and its associated non-histone proteins play an important role in banding formation (Comings *et al.*, 1973; Sumner and Evans, 1973). The significance of the fine type of bands is not clear; however, this type of structure may be the basic configuration upon which the G bands are formed. Although the mechanism of banding in living cells and in fixed preparations may differ, some common feature probably exists since identical patterns result. In fact, fixed chromosome preparations immersed for 1–5 min in a solution of 1 $\mu$g/ml of MNNG in Hanks' medium without $Ca^{++}$ and $Mg^{++}$ and stained with buffered Giemsa also resulted in G bands (unpublished data).

The list of diverse compounds capable of producing chromosome bands now includes chemical carcinogens. It is possible that chromosome bands associated with the carcinogens are a reflection of a nonspecific type of chemical toxicity which leads to cell death because of DNA or associated protein changes that cause conformational changes of nucleoprotein (Meisner *et al.*, 1973). Chromosomal banding may be an immediate response to carcinogen treatment because when the cells were grown for an additional 24 h in medium without carcinogen, no banded metaphases were found. With the cells used in this experiment, partial inhibition of cell multiplication that increased with time occurred after treatment with all carcinogens that caused chromosome bands whereas the non-carcinogens and urethane, neither of which had any effect on cell multiplication, failed to produce bands. Furthermore, urethane treatment actually increased the number of mitoses. In other experiments, the addition of urethane to Syrian hamster cells seeded to form colonies did not result in any transformation or affect colony forming ability (DiPaolo *et al.*, 1972).

Previous studies have shown that the frequency of transformation, a step in the process leading to neoplasia, continues to increase with increasing concentration of chemical carcinogen even after all cells sensitive to the toxic effect of the compound have been killed (DiPaolo *et al.*, 1971*a*). In fact, if the cells are protected from the toxicity of the compounds used in the study, the transformation frequency may even increase (DiPaolo *et al.*, 1971*a*, *b*). Examination of chromosomes of cells transformed by chemical carcinogens reveals that the changes in number of chromosomes or new marker chromosomes, as indicated by banding patterns, are not causally related to the process of carcinogenesis but are reflections of secondary alterations (DiPaolo *et al.*, 1973)

Depending upon the carcinogen concentration used, chromosomal breakage may reflect cell lethality (DiPaolo and Popescu, unpublished data). In the present experiments, breaks and rearrangements occurred following carcinogen treatment which inhibited cell multiplication. The chromosome bands obtained using chemical carcinogens are similar to those reported for compounds such as hydroxyurea and azure B not suspected to be *in vivo* carcinogens. Furthermore, metaphases with chromosome crossbands were not observed with urethane, a well known *in vivo* carcinogen. Therefore, the manifestations reported here probably are not related to transformation, nor can they be expected to serve as an accurate index for surveying chemical compounds for possible carcinogenicity.

### REFERENCES

Caspersson, T., Johanson, C. & Modest, E. (1970) Identification of Human Chromosomes by DNA Binding Fluorescent Agents. *Chromosoma*, **30**, 215.

Comings, D. E., Avelino, E., Okada, T. & Wyandt, H. (1973) The Mechamism of C and G-Banding of Chromosomes. *Expl Cell Res.*, **77**, 469.

DiPaolo, J. A., Donovan, P. J. & Nelson, R. L. (1969) Quantitative Studies of *in vitro* Transformation by Chemical Carcinogens. *J. natn. Cancer Inst.*, **42**, 867.

DiPaolo, J. A., Donovan, P. & Nelson, R. L. (1971*a*) *In Vitro* Transformation of Hamster Cells by Polycyclic Hydrocarbons: Factors Influencing the Number of Cells Transformed. *Nature, New Biol.*, **230**, 240.

DiPaolo, J. A., Donovan, P. & Nelson, R. L. (1971*b*) Transformation of Hamster Cells *in vitro* by Polycyclic Hydrocarbons without Cytotoxicity. *Proc. natn. Acad. Sci. U.S.A.*, **68**, 2958.

DiPaolo, J. A., Nelson, R. L. & Donovan, P. (1972) *In vitro* Transformation of Syrian Hamster Embryo Cells by Diverse Chemical Carcinogens. *Nature, Lond.*, **235**, 278.

DiPaolo, J. A., Popescu, N. C. & Nelson, R. L. (1973) Chromosomal Banding Patterns of Syrian Hamster Cells Transformed *in vitro* by Chemical Carcinogens. *Cancer Res.*, **33**, 3250.

Drets, M. E. & Shaw, M. W. (1971) Specific Banding Patterns of Human Chromosomes. *Proc. natn. Acad. Sci. U.S.A.*, **68**, 2073.

Hsu, T. C., Pathak, S. & Shafer, D. (1973) Induction of Chromosome Cross Banding by Treating Cells with Chemical Agents before Fixation. *Expl Cell Res.*, **79**, 484.

Meisner, L. F., Chuprevich, T. W. & Inhorn, S. L. (1973) Giemsa Banding Specificity. *Nature, New Biol.*, **245**, 145.

Meisner, L. F., Chuprevich, T. W. & Inhorn, S. L. (1973) Chromosome Banding in G2 with Tetracycline. *Lancet*, *i*, 1509.

Popescu, N. C. & DiPaolo, J. A. (1974) Sequential G and C Chromosome Banding. *Lancet*, i, 209.

Rowley, J. D. (1973) A New Consistent Chromosomal Abnormality in Chronic Myelogenous Leukaemia Identified by Quinacrine Fluorescence and Giemsa Staining. *Nature, Lond.*, **243**, 290.

Seabright, M. (1972) The Use of Proteolytic Enzymes for the Mapping of Structural Rearrangements in the Chromosomes in Man. *Chromosoma*, **35**, 204.

Sumner, A. & Evans, H. (1973) Mechanisms Involved in the Banding of Chromosomes with Quinacrine and Giemsa. II. The Interaction of the Dyes with the Chromosomal Components. *Expl Cell Res.*, **31**, 223.

Sumner, A., Evans, H. & Buckland, R. (1971) New Technique for Distinguishing Between Human Chromosomes. *Nature, New Biol.*, **232**, 31.

Yamamoto, T., Rabinowitz, Z. & Sachs, L. (1973) Identification of the Chromosomes that Control Malignancy. *Nature, New Biol.*, **243**, 247.